# Supplementary material for: Comparative Analysis of Comprehensive Genomic Profile in Thymomas and Recurrent Thymomas Reveals Potentially Actionable Mutations for Target Therapies
Source: Int J Mol Sci. 2024 Sep 3;25(17):9560. doi: 10.3390/ijms25179560 (PMC11394945; doi:10.3390/ijms25179560)
Supplement: Supplementary file 1 [file ijms-25-09560-s001.zip › Supplementary Table S1.pdf]

Supplementary Table S1: Comprehensive genome profiling results obtained for the analyzed patients (ID1 – ID37).

Study Group	ID	Gene	Coding change	Aminoacid change	VAF%	Type of alteration	
Rec_Thy	1_P	DIS3	c.1246C>T	p.(Gln416Ter)	53	Nonsense	
	1_R	DIS3	c.1246C>T	p.(Gln416Ter)	48	Nonsense	
		CDK6	-	-	-	Gain	
		CCND3	-	-	-	Gain	
		MDM4	-	-	-	Gain	
	2_P	-	-	-	-	-	
	2_R	DICER1	c.3487_3488del	p.(Lys1163Alafs*3)	13	Frameshift	
	3_P	BRAF	-	-	-	Gain	
		CDK6	-	-	-	Gain	
		FGFR1	-	-	-	Gain	
	3_R	ERCC3	c.1762dup	p.(Glu588Glyfs*16)	42	Frameshift	
	4_P	RAD54L	c.188C>A	p.(Pro63His)	49	Missense	
	4_R	RAD54L	c.188C>A	p.(Pro63His)	49	Missense	
		CDK6	-	-	-	Gain	
		MDM4	-	-	-	Gain	
	5_P	-	-	-	-	-	
	5_R	-	-	-	-	-	
	6_P	MDM4	-	-	-	Gain	
	6_R	MDM4	-	-	-	Gain	
	7_P	-	-	-	-	-	
	7_R	-	-	-	-	-	

	8_P	BRCA1	c.3756_3759del	p.(Ser1253Argfs*10)	8	Frameshift	
		RAD51C	c.414G>C	p.(Leu138Phe)	22	Missense	
	8_R	-	-	-	-	-	
	9_P	-	-	-	-	-	
	10_P	ERCC2	c.361-1G>A	p.(?)	47	Splice site	
	11_R	CCND3	-	-	-	Gain	
		CDK6	-	-	-	Gain	
		CDK4	-	-	-	Gain	
		MDM4	-	-	-	Gain	
	12_P	-	-	-	-	-	
	12_R	CCND3	-	-	-	Gain	
		CDK6	-	-	-	Gain	
		CDK4	-	-	-	Gain	
		MDM4	-	-	-	Gain	
		MYCL	-	-	-	Gain	
	13_R	MDM4	-	-	-	Gain	
	14_P	CCND3	-	-	-	Gain	
		CDK6	-	-	-	Gain	
		MDM4	-	-	-	Gain	
	14_R	-	-	-	-	-	
	15_P	MDM4	-	-	-	Gain	
	15_R	-	-	-	-	-	
	16_P	FGFR4	-	-	-	Gain	
		TP53	c.730G>T	p.(Gly244Cys)	72	Missense	

	16_R	TP53	c.730G>T	p.(Gly244Cys)	79	Missense	
		MYCL	-	-	-	Gain	
	17_P	CDK4	-	-	-	Gain	
	17_R	ARID1B	c.6235C>T	p.(Gln2079Ter)	19	Nonsense	
		MLH1	c.790+1G>T	p.(?)	21	Splice site	
		PARK2/PRKN	c.986dup	p.(Val330Argfs*17)	10	Frameshift	
		SPEN	c.7484dup	p.(Thr2496Tyrfs*2)	16	Frameshift	
		STAG1	c.3558-2A>G	p.(?)	15	Splice site	
		SMC3	c.356del	p.(Asn119Metfs*3)	18	Frameshift	
	18_P	-	-	-	-	-	
	19_P	-	-	-	-	-	
	19_R	CCND3	-	-	-	Gain	
	20_P	IL7R	c.553A>T	p.(Ser185Cys)	56	Missense	
		MDM4	-	-	-	Gain	
	21_P	-	-	-	-	-	
	21_R	MDM4	-	-	-	Gain	
	22_P	-	-	-	-	-	
	22_R	MDM4	-	-	-	Gain	
	23_R	MDM4	-	-	-	Gain	
NoRec_Thy	24	CHEK2	c.470T>C	p.(Ile157Thr)	43	Missense	
		MDM4	-	-	-	Gain	
	25	CDK4	-	-	-	Gain	
	26	MDM4	-	-	-	Gain	
	27	CCND3	-	-	-	Gain	

		CDK6	-	-	-	Gain	
	28	-	-	-	-	-	
	29	ARID1B	c.5458del	p.(His1820Thrfs*18)	6	Frameshift	
		ESR1	c.1181G>A	p.(Arg394His)	50	Missense	
		MYC	-	-	-	Gain	
	30	-	-	-	-	-	
	31	TET2	c.1024G>T	p.(Gly342Ter)	8	Nonsense	
		MDM4	-	-	-	Gain	
	32	PARP1	c.1148_1149delCTinsAA	p.(Ser383Ter)	45	Nonsense	
	33	-	-	-	-	-	
	34	CCND3	-	-	-	Gain	
		CDK6	-	-	-	Gain	
		MDM4	-	-	-	Gain	
	35	CCND3	-	-	-	Gain	
		MEN1	c.485_488del	p.(Val162Glyfs*22)	42	Frameshift	
	36	CDK4	-	-	-	Gain	
		ERCC2	-	-	-	Gain	
		MYCL	-	-	-	Gain	
		NOTCH4	c.4341G>A	p.(Trp1447Ter)	47	Nonsense	
		PIK3CA	c.1258T>C	p.(Cys420Arg)	18	Missense	
	37	CCND3	-	-	-	Gain	
		CDK6	-	-	-	Gain	
		DNMT3A	c.1429+1G>A	p.(?)	7	Splice site	
		MYC	-	-	-	Gain	

		TET2	c.2815C>T	p.(Gln939Ter)	9	Nonsense	
