# Supplementary material for: Comparative Analysis of Comprehensive Genomic Profile in Thymomas and Recurrent Thymomas Reveals Potentially Actionable Mutations for Target Therapies
Source: Int J Mol Sci. 2024 Sep 3;25(17):9560. doi: 10.3390/ijms25179560 (PMC11394945; doi:10.3390/ijms25179560)
Supplement: Supplementary file 1 [file ijms-25-09560-s001.zip › Supplementary Table S2.pdf]

**Supplementary Table S2:** Molecular Targets detected in our analysis (classified as TIER IIC) and the relative clinical trials (ongoing or completed)

| Molecular target | NCT ID (Status) | Study title | Experimental drug [definition] | FDA/EMA-approval |
|---|---|---|---|---|
| CDK4/6 – CCND3 | NCT04116541 (recruiting) | A Study Evaluating the Activity of Anti-cancer Treatments Targeting Tumor Molecular Alterations/Characteristics in Advanced / Metastatic Tumors (MegaMOST) | Ribociclib (plus HDM201) [CDK-inhibitor] | Breast Cancer |
| CDK4/6 – CCND3 | NCT04594005 (active) | CDK4/6 Tumor, Abemaciclib, Paclitaxel | Abemaciclib (plus paclitaxel) [CDK-inhibitor] | Breast Cancer |
| CDK4/6 – CCND3 | NCT03310879 (recruiting) | Study of the CDK4/6 Inhibitor Abemaciclib in Solid Tumors Harboring Genetic Alterations in Genes Encoding D-type Cyclins or Amplification of CDK4 or CDK6 | Abemaciclib [CDK-inhibitor] | Breast Cancer |
| CDK4/6 | NCT02693535 (recruiting) | TAPUR: Testing the Use of Food and Drug Administration (FDA) Approved Drugs That Target a Specific Abnormality in a Tumor Gene in People With Advanced Stage Cancer (TAPUR) | Abemaciclib [CDK-inhibitor] | Breast Cancer |
| CDK4/6 | NCT03239015 (unknown) | Efficacy and Safety of Precision Therapy in Refractory Tumor | Palbociclib [CDK-inhibitor] | Breast Cancer |
| CDK4 | NCT03297606 (recruiting) | Canadian Profiling and Targeted Agent Utilization Trial (CAPTUR) (CAPTUR) | Palbociclib [CDK-inhibitor] | Breast Cancer |
| CDK4/6 | NCT02693535 (recruiting) | TAPUR: Testing the Use of Food and Drug Administration (FDA) Approved Drugs That Target a Specific Abnormality in a Tumor Gene in People With Advanced Stage Cancer (TAPUR) | Palbociclib [CDK-inhibitor] | Breast Cancer |
| BRCA1 – CHEK2 - RAD51C – RAD54L | NCT04123366 (active) | Study of Olaparib (MK-7339) in Combination With Pembrolizumab (MK-3475) in the Treatment of Homologous Recombination Repair Mutation (HRRm) and/or Homologous Recombination Deficiency (HRD)-Positive Advanced Cancer (MK-7339-007/KEYLYNK-007) | Olaparib (plus pembrolizumab) [PARPi] | Ovary, fallopian tubes, peritoneum cancers; breast cancer; pancreatic cancer; prostate cancer |
| BRCA1 – CHEK2 - RAD51C – RAD54L | NCT03742895 (recruiting) | Efficacy and Safety of Olaparib (MK-7339) in Participants With Previously Treated, Homologous Recombination Repair Mutation (HRRm) or Homologous Recombination Deficiency (HRD) Positive Advanced Cancer (MK-7339-002 / LYNK-002) | Olaparib [PARPi] | Ovary, fallopian tubes, peritoneum cancers; breast cancer; pancreatic cancer; prostate cancer |

| | | | | |
|---|---|---|---|---|
| BRCA1 | NCT03239015 (unknown) | Efficacy and Safety of Precision Therapy in Refractory Tumor | Olaparib [PARPi] | Ovary, fallopian tubes, peritoneum cancers; breast cancer; pancreatic cancer; prostate cancer |
| BRCA1 | NCT06065059 (recruiting) | Study to Evaluate TNG348 Alone and With a PARP Inhibitor in Patients With BRCA 1/2 Mutant or HRD+ Solid Tumors | Olaparib (plus TNG348) [PARPi] | Ovary, fallopian tubes, peritoneum cancers; breast cancer; pancreatic cancer; prostate cancer |
| BRCA1 | NCT03297606 (recruting) | Canadian Profiling and Targeted Agent Utilization Trial (CAPTUR) (CAPTUR) | Olaparib [PARPi] | Ovary, fallopian tubes, peritoneum cancers; breast cancer; pancreatic cancer; prostate cancer |
| BRCA1 | NCT01078662 (active) | Open Label Study to Assess Efficacy and Safety of Olaparib in Confirmed Genetic BRCA1 or BRCA2 Mutation Pats | Olaparib [PARPi] | Ovary, fallopian tubes, peritoneum cancers; breast cancer; pancreatic cancer; prostate cancer |
| BRCA1 | NCT02338622 (completed) | Trial of Olaparib in Combination With AZD5363 (ComPAKT) (ComPAKT) | Olaparib (plus AZD5363) [PARPi] | Ovary, fallopian tubes, peritoneum cancers; breast cancer; pancreatic cancer; prostate cancer |
| BRCA1 | NCT02693535 (recruiting) | TAPUR: Testing the Use of Food and Drug Administration (FDA) Approved Drugs That Target a Specific Abnormality in a Tumor Gene in People With Advanced Stage Cancer (TAPUR) | Olaparib [PARPi] | Ovary, fallopian tubes, peritoneum cancers; breast cancer; pancreatic cancer; prostate cancer |
| BRCA1 | NCT02358200 (terminated) | Study of BMN-673 With Carboplatin and Paclitaxel in Patients With Advanced BRCA-mutated Solid Tumor or Triple Negative Metastatic Breast Cancer | Talazoparib (plus paclitaxel/carboplatin) [PARPi] | Breast cancer |

| | | | | |
|---|---|---|---|---|
| BRCA1 – CHEK2 – MLH1 | NCT02693535 (recruiting) | TAPUR: Testing the Use of Food and Drug Administration (FDA) Approved Drugs That Target a Specific Abnormality in a Tumor Gene in People With Advanced Stage Cancer (TAPUR) | Talazoparib (plus atezolizumab) [PARPi] | Breast cancer |
| BRCA1 | NCT05097599 (recruiting) | Strata PATH™ (Precision Indications for Approved Therapies) (Strata PATH) | Talazoparib [PARPi] | Breast cancer |
| BRCA1 | NCT03565991 (terminated) | Javelin BRCA/ATM: Avelumab Plus Talazoparib in Patients With BRCA or ATM Mutant Solid Tumors | Talazoparib (plus avelumab) [PARPi] | Breast cancer |
| BRCA1 | NCT02358200 (terminated) | Study of BMN-673 With Carboplatin and Paclitaxel in Patients With Advanced BRCA-mutated Solid Tumor or Triple Negative Metastatic Breast Cancer | Talazoparib (plus paclitaxel/carboplatin) [PARPi] | Breast cancer |
| BRCA1 – CHEK2 – MLH1 | NCT02693535 (recruiting) | TAPUR: Testing the Use of Food and Drug Administration (FDA) Approved Drugs That Target a Specific Abnormality in a Tumor Gene in People With Advanced Stage Cancer (TAPUR) | Talazoparib (plus atezolizumab) [PARPi] | Breast cancer |
| BRCA1 | NCT04591431 (active) | The Rome Trial From Histology to Target: the Road to Personalize Target Therapy and Immunotherapy (ROME) | Talazoparib [PARPi] | Breast cancer |
| BRCA1 | NCT01482715 (completed) | A Study of Oral Rucaparib in Patients With a Solid Tumor (Phase I) or With gBRCA Mutation Ovarian Cancer (Phase II) | Rucaparib [PARPi] | Ovary, fallopian tubes, peritoneum cancers |
| BRCA1 | NCT01434316 (active) | Veliparib and Dinaciclib in Treating Patients With Advanced Solid Tumors | Veliparib (plus Dinaciclib) [PARPi] | NSCLC (orphan drug) |
| BRCA1 | NCT02210663 (completed) | A Phase 1 Study of Single Agent Veliparib in Japanese Subjects With Advanced Solid Tumors | Veliparib [PARPi] | NSCLC (orphan drug) |
| BRCA1 – CHEK2 - RAD51C – RAD54L | NCT05740956 (recruiting) | A Study of Hansoh (HS)-10502 in Patients With Advanced Solid Tumors | HS-10502 [PARPi] | |
| BRCA1 – CHEK2 - RAD51C – RAD54L | NCT05071209 (active) | Elimusertib for the Treatment of Relapsed or Refractory Solid Tumors | Elimusertib [ATM-kinase inhibitor] | - |
| BRCA1 – CHEK2 - RAD51C – RAD54L | NCT04826341 | A Phase I/II Study of Sacituzumab Govitecan Plus Berzosertib in Small Cell Lung Cancer, Extra-Pulmonary Small Cell Neuroendocrine Cancer and Homologous Recombination-Deficient Cancers Resistant to PARP Inhibitors | Berzosertib [ATR-kinase inhibitor] | - |

| | | | | |
|---|---|---|---|---|
| BRCA1 – CHEK2 - RAD51C – RAD54L | NCT05787587 (recruiting) | A Study of PARG Inhibitor IDE161 in Participants With Advanced Solid Tumors | IDE-161 [PARG- inhibitor] | - |
| BRCA1 – CHEK2 – RAD51C | NCT02873975 (completed) | A Study of LY2606368 (Prexasertib) in Patients With Solid Tumors With Replicative Stress or Homologous Repair Deficiency | Prexasertib [antiCHK1] | - |
| MLH1 - BRCA1 | NCT02693535 (recruiting) | TAPUR: Testing the Use of Food and Drug Administration (FDA) Approved Drugs That Target a Specific Abnormality in a Tumor Gene in People With Advanced Stage Cancer (TAPUR) | Nivolumab (plus ipilimumab) [anti-PD1] | Melanoma, NSCLC, renal cell carcinoma, Hodgkin lymphoma, SCCHN, urothelial cancer, mesothelioma, colorectal, oesophageal cancer |
| PIK3CA | NCT02260661 (completed) | Phase I, Dose Study to Look at the Safety and Pharmacokinetics of AZD8835 in Patients With Advanced Solid Tumours | AZD8835 [PIK3CA inhibitor] | - |
| PIK3CA | NCT05216432 (recruiting) | First-in-Human Study of Mutant-selective PI3K$\alpha$ Inhibitor, RLY-2608, as a Single Agent in Advanced Solid Tumor Patients and in Combination With Fulvestrant in Patients With Advanced Breast Cancer | RLY-2608 [PIK3CA inhibitor] | - |
| PIK3CA | NCT03544905 (recruting) | Study to Evaluate the Safety, Tolerate, Pharmacokinetics and Preliminary Efficacy of CYH33 | CYH33 [PIK3CA inhibitor] | - |
| PIK3CA | NCT04589845 (recruiting) | Tumor-Agnostic Precision Immuno-Oncology and Somatic Targeting Rational for You (TAPISTRY) Platform Study | GDC-0077 (Inavolisib) [PI3K inhibitor] | - |
| PIK3CA | NCT01306045 (active) | Molecular Profiling and Targeted Therapy for Advanced Non-Small Cell Lung Cancer, Small Cell Lung Cancer, and Thymic Malignancies | MK-2206 [AKT inibitor] | - |
| PIK3CA | NCT03297606 (recruting) | Canadian Profiling and Targeted Agent Utilization Trial (CAPTUR) (CAPTUR) | Temsirolimus [mTOR inhibitor] | Renal cell carcinoma, mantle cell lymphoma |
| PIK3CA | NCT03065062 (recruiting) | Study of the CDK4/6 Inhibitor Palbociclib (PD-0332991) in Combination With the PI3K/mTOR Inhibitor Gedatolisib (PF-05212384) for Patients With Advanced Squamous Cell Lung, Pancreatic, Head & Neck and Other Solid Tumors | Gedatolisib (plus palbociclib) [PI3K/mTOR inhibitor] | - |
| PIK3CA | NCT03239015 (unknown) | Efficacy and Safety of Precision Therapy in Refractory Tumor | Everolimus [mTOR inhibitor] | Breast cancer, pancreatic |

| | | | | |
|---|---|---|---|---|
| | | | | neuroendocrine tumours, neuroendocrine tumours, advanced renal cell carcinoma, renal angiomyolipoma and tuberous sclerosis complex |
| PIK3CA | NCT02465060 (active) | Targeted Therapy Directed by Genetic Testing in Treating Patients With Advanced Refractory Solid Tumors, Lymphomas, or Multiple Myeloma (The MATCH Screening Trial) | Taselisib [PIK3CA inhibitor] | - |
| PIK3CA | NCT04591431 (active) | The Rome Trial From Histology to Target: the Road to Personalize Target Therapy and Immunotherapy (ROME) | Ipatasertib [AKT inibitor] | - |
| FGFR1 | NCT03297606 (recruting) | Canadian Profiling and Targeted Agent Utilization Trial (CAPTUR) (CAPTUR) | Sunitinib [RTKs-inhibitor] | Colorectal, GIST, neuroendocrine tumours, |
| FGFR1 | NCT01283945 (completed) | Study of Oral Lucitanib (E-3810), a Dual VEGFR-FGFR Tyrosine Kinase Inhibitor, in Patients With Solid Tumors | Lucitanib [VEGFRs/FGFR inhibitor] | - |
| FGFR1 | NCT02693535 (recruiting) | TAPUR: Testing the Use of Food and Drug Administration (FDA) Approved Drugs That Target a Specific Abnormality in a Tumor Gene in People With Advanced Stage Cancer (TAPUR) | Futibatinib [FGFR inhibitor] | Cholangiocarcinoma |
| FGFR1 | NCT04962867 (active) | NCCH2006/MK010 Trial (FORTUNE Trial) | E7090 [FGF/FGFR inhibitor] | - |
| FGFR1 | NCT04233567 (active) | Infigratinib for the Treatment of Advanced or Metastatic Solid Tumors in Patients With FGFR Gene Mutations | Infigratinib [FGFR inhibitor] | Cholangiocarcinoma |
| FGFR1 | NCT01752920 (completed) | Phase 1/2 Study of Derazantinib (ARQ 087) in Adult Subjects With Advanced Solid Tumors With FGFR Genetic Alterations | Derazantinib [FGFR inhibitor] | - |
| FGFR1 | NCT01948297 (terminated) | Debio 1347-101 Phase I Trial in Advanced Solid Tumours With Fibroblast Growth Factor Receptor (FGFR) Alterations | Debio1347 (zoligratinib) [FGFR inhibitor] | - |

| | | | | |
|---|---|---|---|---|
| FGFR1 | NCT04116541 (recruiting) | A Study Evaluating the Activity of Anti-cancer Treatments Targeting Tumor Molecular Alterations/Characteristics in Advanced / Metastatic Tumors. (MegaMOST) | Regorafenib [TK-inhibitor] | Colorectal cancer; GIST |
| FGFR1 | NCT02465060 (active) | Targeted Therapy Directed by Genetic Testing in Treating Patients With Advanced Refractory Solid Tumors, Lymphomas, or Multiple Myeloma (The MATCH Screening Trial) | Erdafitinib [FGFR inhibitor] | - |
| FGFR1 | NCT04591431 (active) | The Rome Trial From Histology to Target: the Road to Personalize Target Therapy and Immunotherapy (ROME) | Pemigatinib [FGFR inhibitor] | - |
| MYC | NCT03568656 (recruiting) | Study to Evaluate CCS1477 in Advanced Tumours | CCS1477 [p300/CBP bromodomain inhibitor] | - |
| MYC | NCT02873975 (completed) | A Study of LY2606368 (Prexasertib) in Patients With Solid Tumors With Replicative Stress or Homologous Repair Deficiency | Prexasertib [antiCHK1] | - |
| MYC | NCT02635672 (active) | Phase I Dose Escalation Study for VIP152 in Patients With Advanced Cancer | VIP152 [PTEF-b/CDK9 inhibitor] | - |
| MYC | NCT05159518 (completed) | A Study of PRT2527 in Participants With Advanced Solid Tumors | PRT2527 [PTEF-b/CDK9 inhibitor] | - |
| MYC | NCT04872166 (recruiting) | A Study of BTX-A51 in People With Advanced Solid Tumor or Non-Hodgkin Lymphoma | BTX-A51 [CK1alpha/CDK7/CDK9 inhibitor] | - |
| MYC | NCT02656849 (withdrawn) | BAY 1000394 for MCL-1-, MYC-, and CCNE1-Amplified Tumors | Roniciclib [panCDK inhibitor] | - |
| MYC | NCT04983810 (recruiting) | A Study to Investigate Fadraciclib (CYC065), in Subjects With Advanced Solid Tumors and Lymphoma | Fadraciclib [CDK2/5/9 inhibitor] | - |
| MYC | NCT03568656 (recruiting) | Study to Evaluate CCS1477 in Advanced Tumours | CCS1477 [p300/CBP bromodomain inhibitor] | - |
| MYC | NCT02873975 (completed) | A Study of LY2606368 (Prexasertib) in Patients With Solid Tumors With Replicative Stress or Homologous Repair Deficiency | Prexasertib [antiCHK1] | - |
| MYC | NCT02635672 (active) | Phase I Dose Escalation Study for VIP152 in Patients With Advanced Cancer | VIP152 [PTEF-b/CDK9 inhibitor] | - |
