# Supplementary material for: Comparative Analysis of Comprehensive Genomic Profile in Thymomas and Recurrent Thymomas Reveals Potentially Actionable Mutations for Target Therapies
Source: Int J Mol Sci. 2024 Sep 3;25(17):9560. doi: 10.3390/ijms25179560 (PMC11394945; doi:10.3390/ijms25179560)
Supplement: Supplementary file 1 [file ijms-25-09560-s001.zip › Supplementary Table S3.pdf]

Supplementary Table S3. Gene content of the TruSightTM Oncology 500 (TSO500) panel (523 genes - 1.97Mb

ABL1	BTK	DDX41	FANCL	GRM3	IRS2	MLLT3	PDCD1	RAD51B	SOCS1	WISP3
ABL2	C11orf30	DHX15	FAS	GSK3B	JAK1	MPL	PDCD1LG2	RAD51C	SOX10	WT1
ACVR1	CALR	DICER1	FAT1	H3F3A	JAK2	MRE11A	**PDGFRA**	RAD51D	SOX17	XIAP
ACVR1B	CARD11	DIS3	FBXW7	H3F3B	JAK3	MSH2	**PDGFRB**	RAD52	SOX2	XPO1
AKT1	CASP8	DNAJB1	FGF1	H3F3C	JUN	MSH3	PDK1	RAD54L	SOX9	XRCC2
**AKT2**	CBFB	DNMT1	FGF10	HGF	KAT6A	MSH6	PDPK1	**RAF1**	SPEN	YAP1
AKT3	CBL	DNMT3A	FGF14	HIST1H1C	KDM5A	MST1	PGR	RANBP2	SPOP	YES1
**ALK**	**CCND1**	DNMT3B	FGF19	HIST1H2BD	KDM5C	MST1R	PHF6	RARA	SPTA1	ZBTB2
ALOX12B	CCND2	DOT1L	FGF2	HIST1H3A	KDM6A	MTOR	PHOX2B	RASA1	SRC	ZBTB7A
ANKRD11	**CCND3**	E2F3	FGF23	HIST1H3B	KDR	MUTYH	PIK3C2B	RB1	SRSF2	ZFHX3
ANKRD26	**CCNE1**	EED	FGF3	HIST1H3C	KEAP1	MYB	PIK3C2G	RBM10	STAG1	ZNF217
APC	CD274	EGFL7	FGF4	HIST1H3D	KEL	MYC	PIK3C3	RECQL4	STAG2	ZNF703
**AR**	CD276	**EGFR**	FGF5	HIST1H3E	KIF5B	**MYCL1**	PIK3CA	REL	STAT3	ZRSR2
ARAF	CD74	EIF1AX	FGF6	HIST1H3F	**KIT**	**MYCN**	PIK3CB	**RET**	STAT4	
ARFRP1	CD79A	EIF4A2	FGF7	HIST1H3G	KLF4	MYD88	PIK3CD	RFWD2	STAT5A	
ARID1A	CD79B	EIF4E	FGF8	HIST1H3H	KLHL6	MYOD1	PIK3CG	RHEB	STAT5B	
ARID1B	CDC73	EML4	FGF9	HIST1H3I	KMT2B	NAB2	PIK3R1	RHOA	STK11	
ARID2	CDH1	EP300	FGFR1	HIST1H3J	KMT2C	NBN	PIK3R2	**RICTOR**	STK40	
ARID5B	CDK12	EPCAM	FGFR2	HIST2H3A	KMT2D	NCOA3	PIK3R3	RIT1	SUFU	
ASXL1	**CDK4**	EPHA3	FGFR3	HIST2H3C	**KRAS**	NCOR1	PIM1	RNF43	SUZ12	
ASXL2	**CDK6**	EPHA5	FGFR4	HIST2H3D	**LAMP1**	NEGR1	PLCG2	ROS1	SYK	
**ATM**	CDK8	EPHA7	FH	HIST3H3	LATS1	NF1	PLK2	RPS6KA4	TAF1	
ATR	CDKN1A	EPHB1	FLCN	HLA-A	LATS2	NF2	PMAIP1	**RPS6KB1**	TBX3	
ATRX	CDKN1B	**ERBB2**	FLI1	HLA-B	LMO1	NFE2L2	PMS1	RPS6KB2	TCEB1	
AURKA	CDKN2A	**ERBB3**	FLT1	HLA-C	LRP1B	NFKBIA	PMS2	RPTOR	TCF3	
AURKB	CDKN2B	ERBB4	FLT3	HNF1A	LYN	NKX2-1	PNRC1	RUNX1	TCF7L2	
AXIN1	CDKN2C	**ERCC1**	FLT4	HNRNPK	LZTR1	NKX3-1	POLD1	RUNX1T1	TERC	
AXIN2	CEBPA	**ERCC2**	FOXA1	HOXB13	MAGI2	NOTCH1	POLE	RYBP	TERT*	
AXL	CENPA	ERCC3	FOXL2	HRAS	MALT1	NOTCH2	PPARG	SDHA	TET1	

B2M	CHD2	ERCC4	FOXO1	HSD3B1	MAP2K1	NOTCH3	PPM1D	SDHAF2	TET2	
BAP1	CHD4	ERCC5	FOXP1	HSP90AA1	MAP2K2	NOTCH4	PPP2R1A	SDHB	TFE3	
BARD1	**CHEK1**	ERG	FRS2	ICOSLG	MAP2K4	NPM1	PPP2R2A	SDHC	**TFRC**	
BBC3	**CHEK2**	ERRFI1	FUBP1	ID3	MAP3K1	**NRAS**	PPP6C	SDHD	TGFBR1	
BCL10	CIC	**ESR1**	FYN	IDH1	MAP3K13	**NRG1**	PRDM1	SETBP1	TGFBR2	
BCL2	CREBBP	ETS1	GABRA6	IDH2	MAP3K14	NSD1	PREX2	SETD2	TMEM127	
BCL2L1	CRKL	ETV1	GATA1	IFNGR1	MAP3K4	NTRK1	PRKAR1A	SF3B1	TMPRSS2	
BCL2L11	CRLF2	ETV4	GATA2	IGF1	MAPK1	NTRK2	PRKCI	SH2B3	TNFAIP3	
BCL2L2	CSF1R	ETV5	GATA3	IGF1R	MAPK3	NTRK3	PRKDC	SH2D1A	TNFRSF14	
BCL6	CSF3R	ETV6	GATA4	IGF2	MAX	NUP93	PRSS8	SHQ1	TOP1	
BCOR	CSNK1A1	EWSR1	GATA6	IKBKE	MCL1	NUTM1	PTCH1	SLIT2	TOP2A	
BCORL1	CTCF	EZH2	GEN1	IKZF1	MDC1	PAK1	**PTEN**	SLX4	TP53	
BCR	CTLA4	FAM123B	GID4	IL10	**MDM2**	PAK3	PTPN11	SMAD2	TP63	
BIRC3	CTNNA1	FAM175A	GLI1	IL7R	**MDM4**	PAK7	PTPRD	SMAD3	TRAF2	
BLM	CTNNB1	FAM46C	GNA11	INHA	MED12	PALB2	PTPRS	SMAD4	TRAF7	
BMPR1A	CUL3	FANCA	GNA13	INHBA	MEF2B	PARK2	PTPRT	SMARCA4	TSC1	
**BRAF**	CUX1	FANCC	GNAQ	INPP4A	MEN1	PARP1	QKI	SMARCB1	TSC2	
**BRCA1**	CXCR4	FANCD2	GNAS	INPP4B	**MET**	PAX3	RAB35	SMARCD1	TSHR	
**BRCA2**	CYLD	FANCE	GPR124	INSR	MGA	PAX5	RAC1	SMC1A	U2AF1	
BRD4	DAXX	FANCF	GPS2	IRF2	MITF	PAX7	RAD21	SMC3	VEGFA	
BRIP1	DCUN1D1	FANCG	GREM1	IRF4	MLH1	PAX8	RAD50	SMO	VHL	
BTG1	DDR2	FANCI	GRIN2A	IRS1	MLL	PBRM1	RAD51	SNCAIP	VTCN1	

*Promoter region covered only

Copy number alteration calling is available for genes in bold (gain)
